# Unveiling the enigmatic connection: Belly dancer's dyskinesia as an unusual manifestation of normal pressure hydrocephalus: A case report

**DOI:** 10.1002/ccr3.9556

**Published:** 2024-11-03

**Authors:** Sunil Thatal, Susmin Karki, Asmita Parajuli, Sweta Bhandari, Sagar Mani Regmi, Navin Kumar Sah

**Affiliations:** ^1^ B.P. Koirala Institute of Health Sciences Dharan Nepal; ^2^ Maharajgunj Medical Campus Tribhuvan University Institute of Medicine Kathmandu Nepal; ^3^ Department of Internal medicine B.P. Koirala Institute of Health Sciences Dharan Nepal

**Keywords:** abdominal myoclonus, belly dancer's dyskinesia, normal pressure hydrocephalus, NPH

## Abstract

**Key Clinical Message:**

Normal pressure hydrocephalus is rarely associated with Belly dancer's dyskinesia and seizure.

**Abstract:**

Belly dancer's dyskinesia (BDD) is characterized by bilateral, sluggish, involuntary, repetitive, and rhythmic motions of the anterior abdominal wall. We present a rare case of a 78‐year‐old man diagnosed with normal pressure hydrocephalus associated with BDD and seizure who presented with left‐sided weakness of the body and abnormal body movements.

## 1 INTRODUCTION

In 1723, Anthonie Van Leeuwenhoek, who himself was a sufferer of belly dancer's dyskinesia (BDD), coined the term diaphragmatic flutter (DF) for abdominal myoclonus. Later, in 1990, it was first reported as a rare condition, renamed “Belly dancer's dyskinesia” due to its resemblance to the movements of a belly dancer.^1^, ^2^ Although mentioned in various literature, the exact pathophysiologic mechanism of BDD is yet to be concretely described.^2^ Normal pressure hydrocephalus (NPH) is a treatable, reversible disorder with the classic triad of magnetic ataxia, urinary incontinence, and dementia.^3^ Seizures and abdominal myoclonus, as such, are rare findings in NPH. No literature explaining the association between NPH, and abdominal myoclonus was found, suggesting it to be a rare association. Here, we present a rare case of a 78‐year‐old male diagnosed with NPH showing atypical association with abdominal wall myoclonus and seizure.

## 2 CASE HISTORY AND EXAMINATION

A 78‐year‐old man who is a known case of type 2 diabetes mellitus under oral hypoglycemics presented to the emergency with complaints of left‐sided weakness of the body for 3 days and one episode of abnormal body movement. The patient developed left‐sided weakness, more in the left arm than the leg, which was insidious in onset, gradually progressive, and associated with pain and numbness along the left arm. The abnormal body movements were acute in onset and characterized by generalized tonic–clonic seizure with up rolling of eyes and deviation of mouth. It was also associated with involuntary passage of urine and altered sensorium. The patient had similar 3–4 episodes of abnormal body movements 6 months back. The patient also complained of abnormal abdominal movements, which were characterized by involuntary, rhythmic, and repetitive movements that were not present during sleep (Video 1). There was no history of fever, vomiting, loss of consciousness, pain in the abdomen, trauma, decreased vision, and bowel and bladder incontinence.

**VIDEO 1:** The patient is moaning throughout the video.

On examination, the patient was not oriented to time, place, and person. His vitals were stable. Other systemic examinations were within normal limits. There were no signs of meningeal irritation, and cranial nerve examinations were within normal limits. His motor examination revealed a power of ⅖ in the left upper and ⅗ in the left lower limb, while the right side of the body had a power of 5/5, according to the Medical Research Council (MRC) scale. He had decreased sensation over the left half of the body compared to the right. His reflexes over major joints were decreased on the left side compared to the right side. His bilateral plantar was down going. Fundoscopy showed no signs of increased intracranial pressure.

## 3 METHODS

**FIGURE 1:**
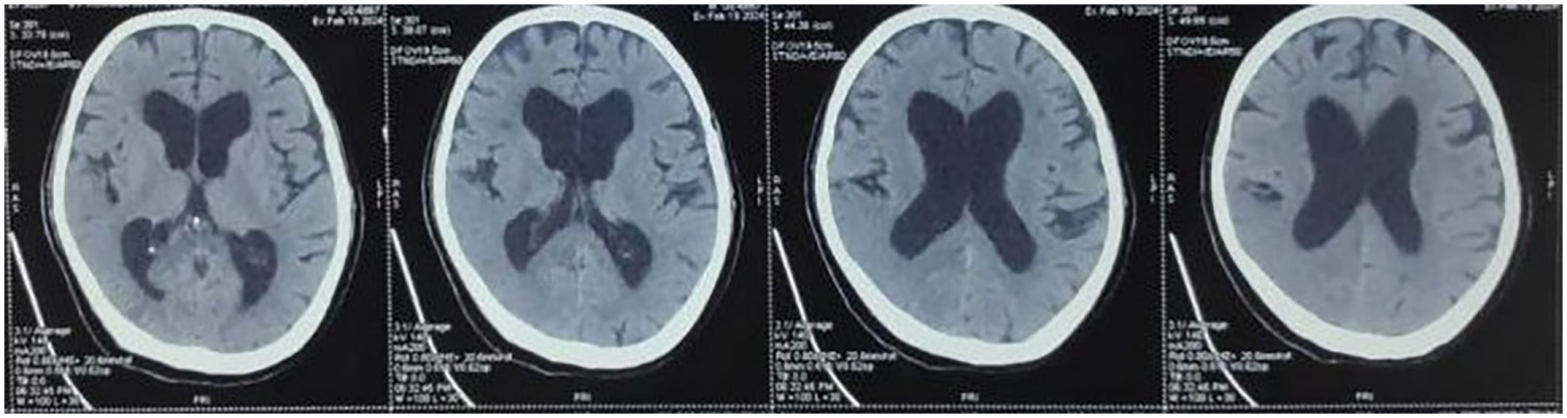
The CT head showing dilated lateral and third ventricle with mildly prominent bilateral sylvian fissures, cortical sulci, ventricular system, basal cisterns, and cerebellar folia suggestive of diffuse brain atrophy.

**FIGURE 2:**
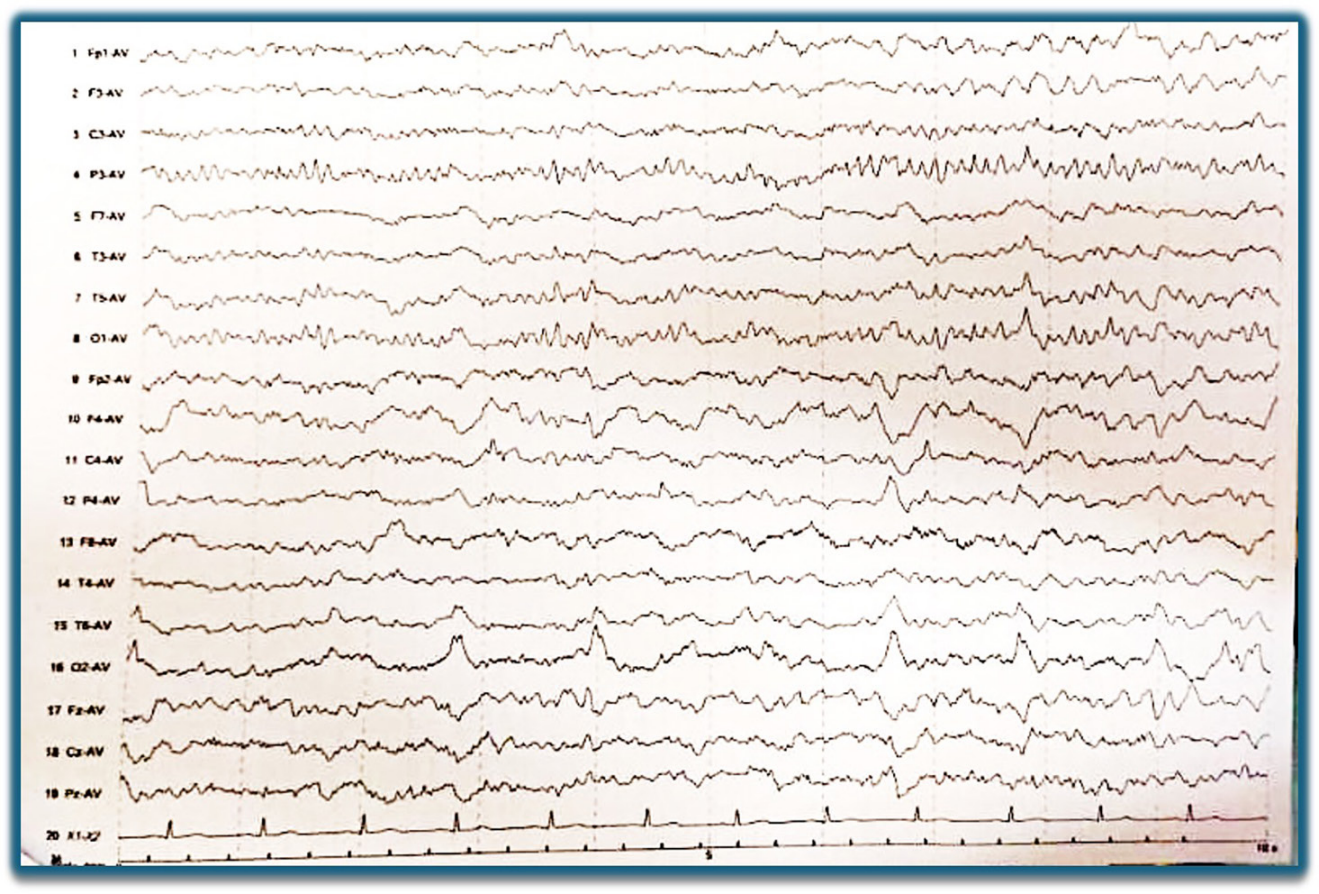
Waking state electroencephalogram (EEG) showing hemispheric asymmetry and severe right hemispheric encephalopathic changes along with epileptiform discharges.

A provisional diagnosis of acute stroke was made, for which the patient was transferred for emergency computed tomography (CT) of the head. The CT head showed dilated lateral and third ventricles with mildly prominent bilateral sylvian fissures, cortical sulci, ventricular system, basal cisterns, and cerebellar folia suggestive of diffuse brain atrophy (Figure 1). Waking state electroencephalogram (EEG) showed hemispheric asymmetry and severe right hemispheric encephalopathic changes along with epileptiform discharges (Figure 2). A lumbar puncture was done to find out the cause of hydrocephalus, which showed an opening pressure of 10 mm of Hg, with sterile cerebrospinal fluid (CSF) culture. The diagnosis of normal pressure hydrocephalus (NPH) associated with seizure and belly dancer's dyskinesia was made.

The patient was treated with intravenous levetiracetam, but the patient did not respond and then was shifted to intravenous valproic acid. Then, gradually, the generalized tonic–clonic seizure, weakness, along with the abdominal myoclonus subsided. On discharge, the patient had normal neurological examinations. The patient was followed up in the outpatient department (OPD) and found to have normal neurological status.

## 4 CONCLUSION AND RESULTS

In a low‐resource setting, it is challenging to diagnose BDD. Although rare, Belly dancer's dyskinesia and seizure might be associated with NPH. Being a less researched area, its diagnosis and management are done on a case basis, making it more difficult to diagnose and manage in low resource setting. More in‐depth study is warranted regarding this matter.

## 5 DISCUSSION

Belly dancer's dyskinesia (BDD), a rare condition, was first described by Anthonie Van Leeuwenhoek who suffered from the disorder himself and described it as a violent movement of the diaphragm.^1^ Numerous causes, including central nervous system disorders (both organic and psychological), peripheral nervous system disorders, pleural disorders, cardiac disorders, mediastinal disorders, cervical spinal disorders, drug‐induced, and idiopathic causes, have been documented in the literature.^4^ Common drugs leading to BDD include levodopa (dopamine‐regulating medication), quetiapine and prochlorperazine (antidopaminergic), domperidone, and salbutamol.^5^, ^6^, ^7^ There have been reports of drug side effects, vitamin B12 deficiency, delivery, abdominal surgery, intramedullary thoracic cord tumors, osmotic demyelination, encephalitis, basal ganglia lesions, and epilepsy as causes of abdominal myoclonus.^5^, ^8^, ^9^, ^10^, ^11^, ^12^ We couldn't find any mentioned etiologies, but our case was associated with NPH, which might be a chance or causality.

There is usually the gradual onset of belly dancer's dyskinesia and contractions of muscles like rectus abdominis, oblique muscles, paraspinal and perineal muscles are involved. The pathophysiology remains still unclear, though some theories explain the dysfunction of inhibitory spinal interneurons or structural reorganization of local neuronal circuits, which are mainly responsible for these abnormal body movements.^8^ The central origin of BDD usually remains during sleep, whereas the peripheral or spinal origin subsides during sleep. The presence of symptoms only in awakened patients suggests the psychogenic factors responsible for BDD.^13^ In such cases, distracting the patients and breath‐holding may help in the diagnosis of BDD.^14^ Although the clinical features of this unusual movement disease vary significantly, they often involve aberrant jerking movements caused by involuntary contractions of the abdominal wall muscles. Most patients with this uncommon dyskinesia are bilateral and exhibit sluggish, writhing in addition to involuntary, repetitive, occasionally painful, and frequently rhythmic motions of the anterior abdominal wall.^15^ Numerous cases with an extensive list of underlying reasons have been recorded in the literature. However, the precise underlying pathology remains unclear.

Though the diagnosis and management of BDD is difficult and challenging, fluoroscopy and electromyography may aid in its diagnosis. However, these tests still need to be standardized; using them for diagnosis is still a debatable topic. Imaging of the brain and spinal cord can be done to distinguish BDD from other secondary causes.^5^ We performed several investigations to rule out the causes, but we could find none. Being in a resource‐limited setting, our diagnosis was based more on the clinical background.^16^

Most treatments focus on treating the cause or are mostly symptomatic. Drugs like diazepam, haloperidol, aripiprazole, and clonazepam have been used successfully to treat this disorder.^5^, ^14^, ^17^ In some cases, clonazepam has even completely reversed the symptoms of diaphragmatic flutter. When medical treatment fails, surgical methods like phrenic nerve block, transection, or crushing can decrease the symptoms and provide instantaneous relief of symptoms.^15^ Ultrasound guided botulinum toxin A injection has been administered with successful results in a cohort of patients. Hence, no single modality has been recommended for the management of BDD, making its prognosis highly unpredictable.^18^ Symptomatic treatment is the sole course of action in these situations. Most of these cases involve incomplete recovery.^5^ A study by KC et al. showed that the patient responded to intravenous levetiracetam,^19^ whereas, in our case, injection levetiracetam did not do the same; hence, we shifted to valproic acid, which successfully resolved the symptoms of abdominal myoclonus.

## AUTHOR CONTRIBUTIONS

**Sunil Thatal:** Conceptualization; data curation; formal analysis; investigation; methodology; project administration; resources; software; validation; visualization; writing – original draft; writing – review and editing. **Susmin Karki:** Conceptualization; data curation; formal analysis; investigation; methodology; project administration; resources; software; validation; visualization; writing – original draft; writing – review and editing. **Asmita Parajuli:** Conceptualization; data curation; formal analysis; investigation; methodology; project administration; resources; software; validation; visualization; writing – original draft; writing – review and editing. **Sweta Bhandari:** Conceptualization; data curation; formal analysis; investigation; methodology; project administration; resources; software; validation; visualization; writing – original draft; writing – review and editing. **Sagar Mani Regmi:** Conceptualization; data curation; formal analysis; investigation; methodology; project administration; resources; software; validation; visualization; writing – original draft; writing – review and editing. **Navin Kumar Sah:** Conceptualization; data curation; formal analysis; funding acquisition; investigation; methodology; project administration; resources; supervision; writing – review and editing.

## FUNDING INFORMATION

None.

## CONFLICT OF INTEREST STATEMENT

The author(s) declare(s) that there is no conflict of interest regarding the publication of this paper.

## ETHICS STATEMENT

Our institution does not require ethical approval to report individual cases.

## CONSENT

Written informed consent was obtained from the patient to publish this report in accordance with the journal's patient consent policy.

## Data Availability

The data that support the findings of this study are available from the corresponding author, upon reasonable request.
